# Severe Bradycardia and Shock With Delayed Onset Due to Lacosamide Overdose: A Case Report

**DOI:** 10.7759/cureus.79381

**Published:** 2025-02-20

**Authors:** Akifumi Okamoto, Toru Someno, Mariko Okada, Shinya Suzuki

**Affiliations:** 1 Department of Pharmacy, Yokohama City Minato Red Cross Hospital, Yokohama, JPN; 2 Department of Emergency and Critical Care Medicine, Yokohama City Minato Red Cross Hospital, Yokohama, JPN; 3 Center for Experiential Pharmacy Practice, School of Pharmacy, Tokyo University of Pharmacy and Life Sciences, Hachioji, JPN

**Keywords:** bradycardia, lacosamide, overdose, shock, trazodone

## Abstract

Lacosamide is a distinctive antiepileptic drug that exerts its antiepileptic effects by promoting the slow inactivation of voltage-gated sodium channels in the central nervous system. In cases of overdose, it may also affect cardiac sodium channels, leading to conduction disorders. Seizures are common in overdose cases, and severe cases may present with ventricular tachycardia and cardiac arrest. However, severe bradycardia and shock as primary manifestations of overdose are rare. We report a case of multiple-drug overdose, primarily involving lacosamide, associated with delayed onset of severe bradycardia and shock. A 68-year-old woman with Parkinson’s disease (PD) was admitted to the emergency department (ED) with unexplained impaired consciousness. Approximately 8.5 hours after hospital admission, her heart rate suddenly dropped, and she developed shock. Continuous intravenous dopamine infusion was initiated for severe bradycardia and shock, eventually reaching a dose of 10.5 µg/kg/min. Approximately 12.5 hours after admission, the patient’s sister discovered empty packages of several medications at the patient’s home, including approximately 3,350 mg of lacosamide and approximately 1,200 mg of trazodone. Consequently, the patient was diagnosed with drug poisoning. As her condition gradually improved, the dopamine infusion was discontinued on hospital day (HD) 3. After adjusting PD medication, she was transferred to another hospital on HD 72. Serum lacosamide concentration was elevated to 91.5 µg/mL approximately 8.5 hours after hospital admission, consistent with the onset of bradycardia and shock. This case highlights the need for careful monitoring of patients with lacosamide overdose owing to the potential for delayed severe bradycardia and shock.

## Introduction

Lacosamide is a distinctive antiepileptic drug that exerts antiseizure effects by promoting the slow inactivation of voltage-gated sodium channels [1]. It is primarily metabolized by CYP2C19 in the liver to O-desmethyl-lacosamide, which has no pharmacological activity [2]. The drug has a half-life of approximately 13 hours, and most of it is excreted unchanged in the urine [2]. Common toxidromes include vomiting, seizures, coma, and drowsiness [3]. In severe cases, cardiac arrest and fatal dysrhythmia, such as ventricular tachycardia, have been reported, though severe bradycardia and shock remain rare [4,5].

At high doses, lacosamide can affect cardiac sodium channels, potentially causing dose-dependent conduction disorders and dysrhythmia [6]. However, some cases of serious dysrhythmias linked to lacosamide overdose can occur even with normal serum concentrations, leaving it unclear whether severe bradycardia and shock are serum concentration-dependent [4,5].

We present a case of a multiple-drug overdose, primarily involving lacosamide, with delayed onset of severe bradycardia and shock, including continuous serum lacosamide concentration measurements.

## Case presentation

A 68-year-old woman (53 kg) with Parkinson’s disease (PD) was found comatose in her kitchen by her food delivery person, who alerted emergency medical services (EMS) at 17:49. The day-service staff reported that the patient was in good health until noon that day. Upon EMS arrival at 17:51, the patient’s Glasgow Coma Scale (GCS) score was 3 (E1V1M1), respiratory rate 22 breaths per minute, blood pressure 172/82 mmHg, body temperature 37.5 °C, and oxygen saturation 95% on room air. EMS personnel searched the scene for empty medication packages but found none.

On admission to the emergency department (ED) at 18:27, the patient’s GCS score remained at 3 (E1V1M1), and her condition was unchanged. Vital signs were as follows: respiratory rate 22 breaths per minute, blood pressure 178/86 mmHg, heart rate 72 beats per minute, and oxygen saturation 92% on room air. A 7-mm nasopharyngeal airway was inserted in the right nostril to address oxygen desaturation owing to glossoptosis caused by impaired consciousness. After nasopharyngeal airway insertion, oxygen saturation remained ＞95%, so intubation was not performed. Venous blood gas analysis showed slight respiratory acidosis, but blood biochemistry and complete blood count showed no major abnormalities (Table 1).

**Table 1:** Laboratory test and venous blood gas analysis results of the patient

Laboratory test and venous blood gas analysis	Result	Reference value
White blood cells (×10^3^/μL)	13.0	35.0-91.0
Red blood cells (×10^4^/μL)	396	376-500
Hemoglobin (g/dL)	12.1	11.3-15.2
Platelets (×10^3^/μL)	220	130-36.9
Blood glucose (mg/dL)	130	79-109
Albumin (g/dL)	3.5	4.1-5.1
Blood urine nitrogen (mg/dL)	15.5	8-20
Serum creatinine (mg/dL)	0.61	0.47-0.79
Serum sodium (mEq/L)	138	136-147
Serum potassium (mEq/L)	4.1	3.6-5.0
Total bilirubin (mg/dL)	0.4	0.2-1.0
Aspartate aminotransferase (U/L)	10	10-40
Alanine aminotransferase (U/L)	4	5-40
NH_3_ (µg/dL)	20	12-60
pH	7.309	7.31-7.41
pO_2_ (mmHg)	40.1	30-40
pCO_2_ (mmHg)	54.5	41-51
HCO_3_^-^ (mmol/L)	27.3	18-23
Lactate (mmol/L)	2.8	0.4-2.2

**Figure 1:**
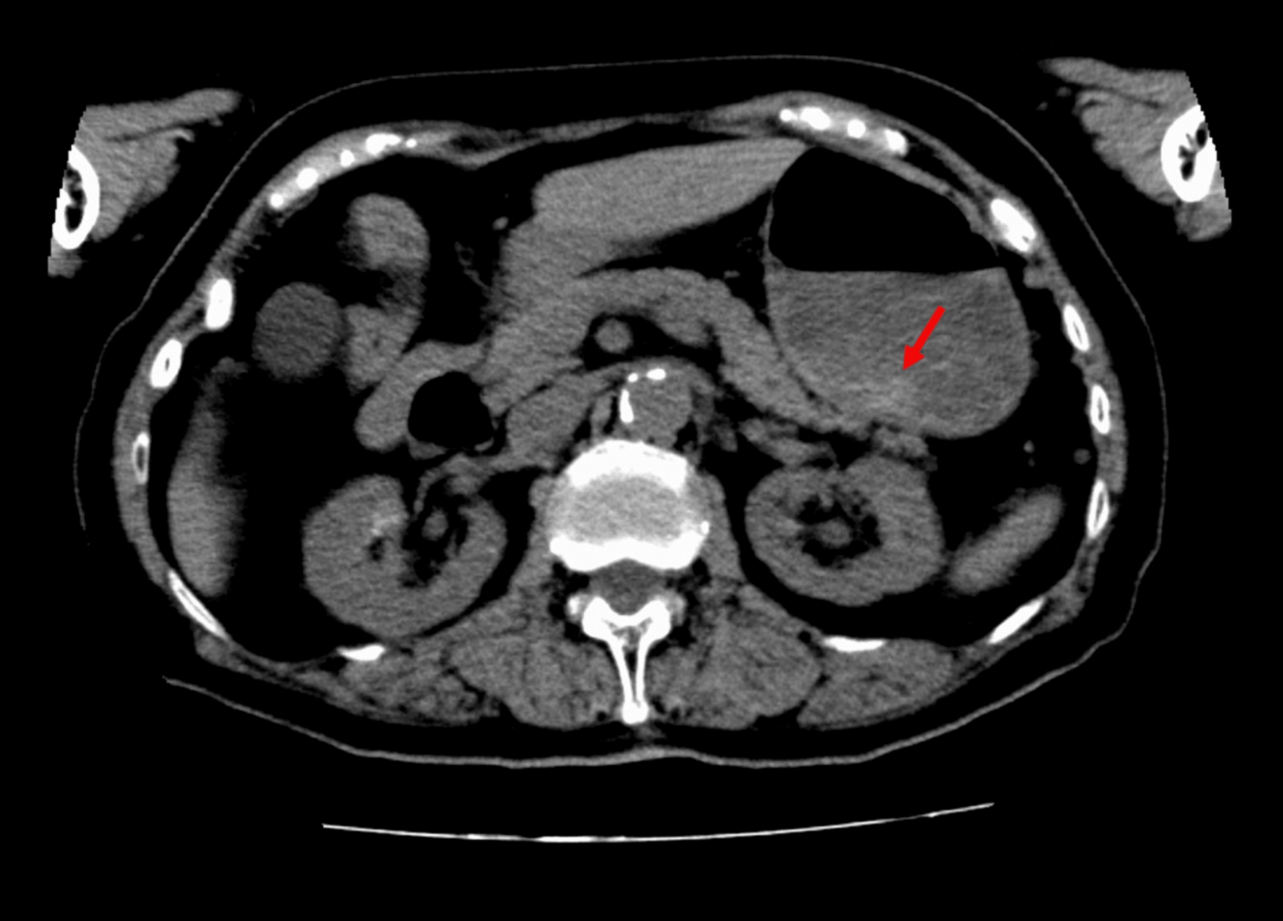
Abdominal CT image after admission The red arrow indicates an area of slightly elevated density in the stomach.

A urine drug screen detected tricyclic antidepressants and benzodiazepines. A computed tomography (CT) scan of the abdomen revealed an area of slightly elevated density in the stomach (Figure 1); however, there was no initial evidence of drug overdose. A CT scan of the head revealed no lesions to explain the coma. Magnetic resonance imaging of the head was not feasible owing to the presence of an intracranially implanted deep brain stimulation (DBS) device for PD treatment. For further evaluation of coma etiology, a lumbar puncture was performed at 21:44. Cerebrospinal fluid analysis revealed no abnormalities, effectively excluding meningitis and encephalitis.

**Figure 2:**
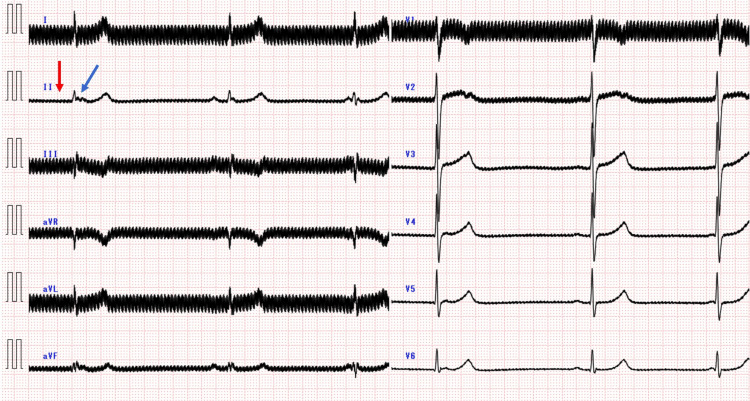
Twelve-lead ECG 8.5 hours after arrival The ECG waveform was significantly noisy owing to interference from the DBS device. Severe bradycardia due to the sinus pause was observed (red arrow). An atrioventricular junctional escape rhythm (blue arrow) was observed after the sinus pause. DBS: deep brain stimulation.

The patient was admitted to the emergency ward at 00:34 on day 2. The patient was hemodynamically stable until 02:57, at which point her heart rate suddenly dropped to 35 beats per minute and her blood pressure to 86/54 mmHg. An electrocardiogram (ECG) performed at symptom onset showed a noisy waveform due to interference from the DBS device. A noisy waveform was already present on the ECG from the time of admission, but there was no prolongation of the PR, QRS, or QTc intervals or bradycardia. The noise was especially prominent in leads I, III, aVR, aVL, and V1 but was legible in lead II. The PR interval was 0.232 seconds, the corrected QT (QTc) interval was 0.504 seconds, and the QRS interval was prolonged to 0.134 sec (Figure 2).

**Figure 3:**
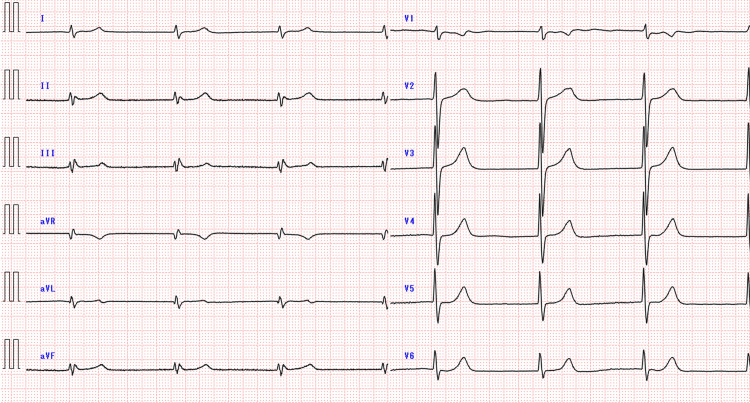
Twelve-lead ECG 19 hours after arrival The noise in the ECG waveform disappeared when the DBS device was turned off. Bradycardia due to the atrioventricular junction rhythm was observed.

Her heart rate decreased to 20-30 beats per minute for approximately 10 minutes. By 03:16, bigeminal premature ventricular contractions occurred, with the heart rate increasing to 40-50 beats per minute and blood pressure remaining at 60/40 mmHg. A cardiologist was consulted for bradycardia and shock. At this point, the cause of the sudden onset of bradycardia and shock was not determined to be an overdose of drugs, and no consultation was made with a medical toxicologist or poison center. Transthoracic echocardiography was performed, and the left ventricular ejection fraction showed normal contraction. Intravenous atropine (0.5 mg) and dopamine (6 mg) were administered at 04:07, followed by continuous dopamine infusion at 5.3 µg/kg/min. Systolic blood pressure improved to 80-90 mmHg, but her heart rate remained 40-50 beats per minute. The dopamine dose was increased to 10.5 µg/kg/min at 06:39 because of persistent bradycardia and shock. At approximately 07:00, the patient’s sister found empty packages of lacosamide (3,350 mg), trazodone (1,300 mg), alprazolam (11.6 mg), and mirtazapine (480 mg) in the patient’s room. The prescription record showed that these drugs were prescribed to the patient herself. She was also prescribed mirtazapine, lemborexant, and levodopa, but empty packages for these drugs were not found in the patient's room. The patient was diagnosed with bradycardia and shock due to an overdose of these medications. With the DBS device deactivated, ECG showed an atrioventricular junctional rhythm with a heart rate of 40-50 beats per minute, a QTc interval of 0.519 seconds, and a QRS interval of 0.128 seconds (Figure 3).

Temporary pacing catheter insertion was considered but withheld as the patient responded to continuous dopamine infusion. In addition, sodium bicarbonate was not administered. By day 3, bradycardia and shock had resolved, allowing the dopamine infusion to be tapered off between 09:23 and 14:58. The patient later admitted to a suicide attempt by overdosing on prescription medications but could not recall the specific types or dosages. Following an adjustment of her PD medications, she was transferred to another hospital on day 72.

**Figure 4:**
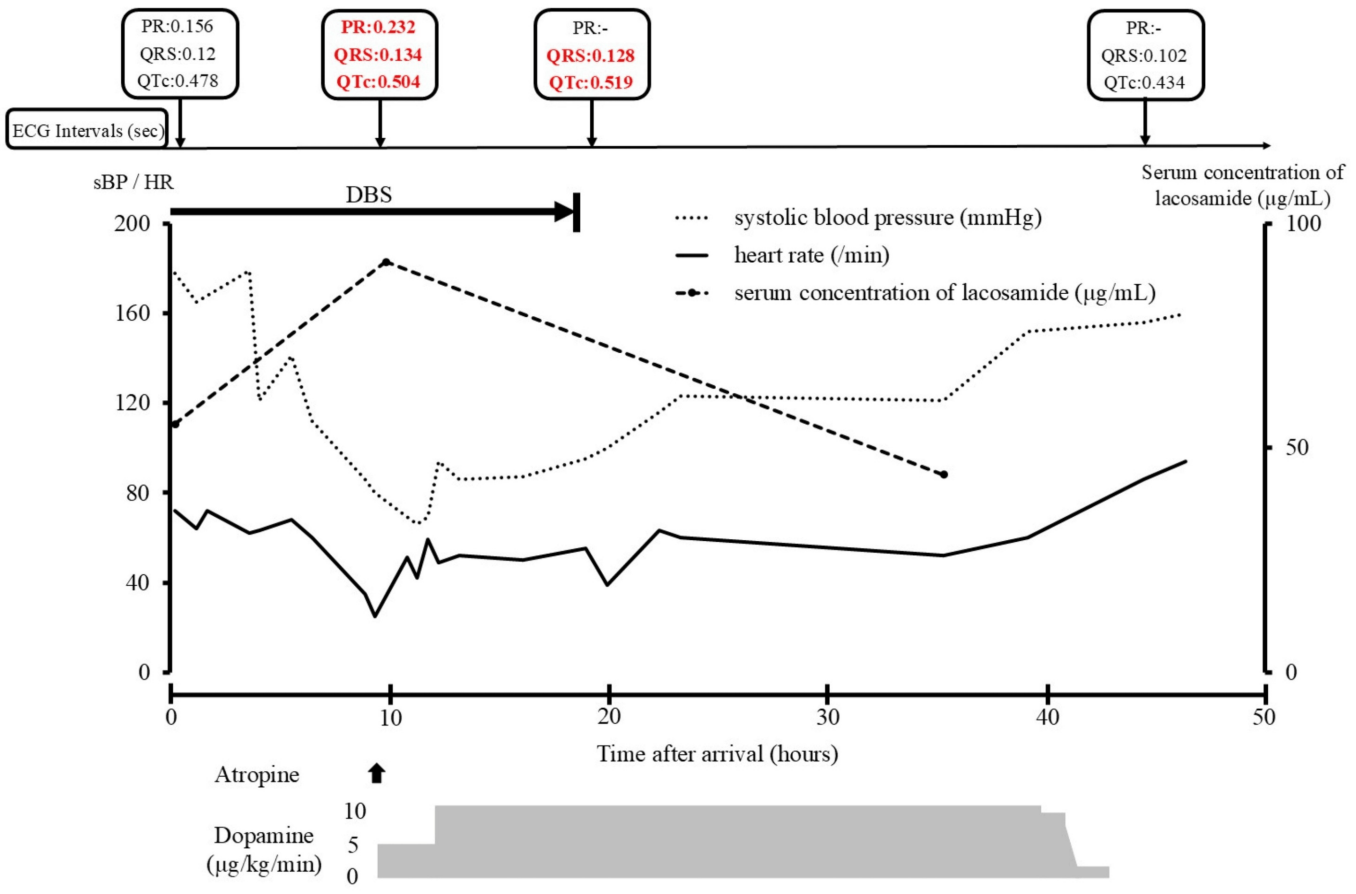
Serial changes in serum lacosamide concentration, heart rate (HR), and systolic blood pressure (sBP), and a timeline of ECG changes Abnormal ECG values are indicated in red text.

Serum lacosamide concentrations were measured via liquid chromatography-tandem mass spectrometry. At admission, the serum lacosamide concentration was 55.4 μg/mL. Subsequent serum samples collected 8.5 hours later showed an increase to 91.5 μg/mL (Figure 4).

## Discussion

In this case, a lacosamide overdose induced severe bradycardia and shock, with continuous serum concentration measurements revealing a concentration-dependent relationship.

Severe lacosamide overdose commonly results in central nervous system toxicity, including seizures and impaired consciousness, as well as tachyarrhythmias such as ventricular tachycardia [4,5,7-11]. While lacosamide exhibits a higher affinity for neuronal sodium channels than cardiac sodium channels, high concentrations affect cardiac sodium channels, leading to ECG changes such as prolonged PR, QRS, and QTc intervals [5-7,9,10]. Takai et al. [10] reported a case in which a fatal dysrhythmia occurred owing to an overdose of lacosamide, and the patient was managed using an extracorporeal membrane oxygenation device. The serum concentration of lacosamide in this case was 153 µg/mL, much higher than in the case presented herein and, to the best of our knowledge, was the highest serum concentration reported to date. Therefore, it is expected that the risk of fatal dysrhythmia increases in proportion to the serum concentration of lacosamide. On the other hand, Malissin et al. [5] reported a case of death due to cardiac arrest, distributive shock, and multiple organ failure caused by an overdose of approximately 7 g of lacosamide, but the serum lacosamide concentration at that time was only slightly above the therapeutic range at 22.7 µg/mL. In this case, the concomitant medications included sodium channel blockers such as carbamazepine and lamotrigine. Even if the serum concentration of lacosamide is not high, it is thought that the toxicity to the heart may have been enhanced by the concomitant use of drugs that exert the same pharmacological effects as lacosamide, and it is expected that caution is needed with concomitant medications.

In the present case, the primary manifestations were bradycardia with prolonged PR, QRS, and QTc intervals, accompanied by transient nonsustained ventricular tachycardia. Bradycardia with atrioventricular block is uncommon in overdose but has been reported with therapeutic doses, particularly following intravenous administration or in older patients [12,13]. The cardiologist treated these symptoms using only atropine and dopamine but, at the time, did not consider administering sodium bicarbonate, as there was no evidence implicating lacosamide in the bradycardia and shock at the time of consultation. In addition, the cardiologist was unfamiliar with the treatment of lacosamide poisoning. Sodium bicarbonate is sometimes administered as an antidote for ventricular dysrhythmia caused by poisoning with some sodium channel blockers, such as tricyclic antidepressants. It works by increasing the number of open Na channels, partially reversing fast Na channel blockage and reducing QRS prolongation. In our case, sodium bicarbonate should have been considered once lacosamide poisoning was strongly suspected. If treatment uncertainty arises, consulting a poison center promptly is essential. Our patient had prolonged QRS, and sodium bicarbonate may have reduced cardiotoxicity. Although several cases of sodium bicarbonate administration for lacosamide poisoning have been reported [4,5,7], its effectiveness in treating cardiotoxicity remains controversial. Malissin et al. [5] reported a sustained reduction in QRS width after administration of sodium bicarbonate in a patient with wide QRS tachycardia, whereas Chua-Tuan et al. [4] and Ng et al. [7] observed no such effect. Since bradycardia and shock seen in our case are atypical symptoms of lacosamide poisoning, we believe dopamine can effectively manage them. For dysrhythmia accompanied by QRS prolongation, sodium bicarbonate should be actively considered, but for bradycardia and shock, continuous infusion of dopamine, not just sodium bicarbonate, should also be considered as an option. Fortunately, our patient stabilized with dopamine alone, avoiding the need for temporary pacing. However, if bradycardia and shock persist despite treatment, early introduction of temporary pacing should be considered. In addition, repeated ECG monitoring is necessary to assess treatment effectiveness.

The reference serum lacosamide concentration is 10-20 µg/mL [14]; however, the peak serum concentration in this case was significantly higher at 91.5 µg/mL. The onset of severe bradycardia and shock, coinciding with prolonged PR, QRS, and QTc intervals, occurred alongside elevated serum lacosamide levels, indicating a dose-dependent effect. Cases with lacosamide serum concentrations similar to ours include those reported by Ng et al., Marcellino et al., and Takahashi et al., with concentrations of 98.4 μg/mL, 80.7 µg/mL, and 91.7 µg/mL, respectively [7,8,11]. While all three cases involved seizures, the only dysrhythmia reported was ventricular tachycardia by Ng et al. and Marcellino et al., with no instances of bradycardia [7,8]. Additionally, these cases lacked a history of arrhythmia, hypotension, or concomitant drug use that could exacerbate bradycardia or shock. The exact cause of the severe bradycardia and shock in our case remains unclear but may be attributed to concomitant medications. Trazodone, in particular, is known to cause central nervous system depression, QT prolongation, and hypotension [15]. Its effects on cardiac conduction are thought to result from inhibition of the human ether-á-go-go-related gene (hERG) channel current, while hypotension is attributed to alpha-adrenergic antagonism [16,17]. hERG is related to the rapidly activating delayed rectifier potassium channel current (IKr), which plays an important role in ventricular repolarization, and it is known that trazodone inhibits this and causes QT prolongation. Soe et al. [18] reported bradycardia and QTc prolongation in patients who ingested up to 4500 mg of trazodone. Regarding the shock, it was considered that it occurred owing to the synergistic effect of bradycardia caused by lacosamide and trazodone, in addition to the decrease in peripheral vascular resistance caused by the alpha-adrenergic antagonistic effect of trazodone. Camacho et al. [17] reported severe hypotension in patients who ingested up to 2,500 mg of trazodone. In our case, the patient possibly ingested 1,300 mg of trazodone in combination with lacosamide; however, this was not possible because there was no equipment in the hospital that could measure the serum concentration of trazodone, and there was no company that could be commissioned to measure it. While the trazodone dose was below its reported toxic threshold when taken alone, its combination with lacosamide may have exacerbated the symptoms. In our case, severe bradycardia likely resulted from the combined effect of trazodone-induced QT prolongation via IKr inhibition and lacosamide-induced cardiac sodium channel blockade. The patient was being prescribed levodopa for PD but was not being prescribed any drugs that could potentially cause QT prolongation or hypotension, such as dopamine agonists or amantadine. Drugs that cause QT prolongation and hypotension, such as trazodone, may potentiate the effects of lacosamide overdose, increasing the risk of severe bradycardia or shock.

Lacosamide is rapidly absorbed after oral administration, reaching maximum serum concentration (Cmax) within 0.5-4 hours [2]. In this case, the time of overdose was unknown, but lacosamide levels increased later than expected. Delayed toxicity is a recognized phenomenon in drug overdose, attributed to factors such as delayed absorption, altered distribution, metabolic changes, and organ dysfunction [19]. In general, the elimination of drugs in cases of overdose often follows zero-order kinetics. In our case, it is possible that the metabolic enzyme CYP2C19, which metabolizes lacosamide, became saturated and that metabolism was delayed. This was evidenced by the calculated half-life of lacosamide, based on serum concentrations at two time points, which was 25 hours longer than the reference value. As a result, it is possible that lacosamide accumulated in the body and caused toxicity. In addition, the delayed absorption caused by an overdose may also have contributed to the appearance of delayed toxicity. Delayed drug absorption can be attributed to the formation of drug bezoars, which are large masses of semi-dissolved products [19]. While no clear evidence of bezoars was observed on the initial CT scan in this case, an area of slightly elevated-density area was observed in the stomach. Therefore, it is possible that the delay in the dissolution of the tablets in the stomach occurred due to the large number of tablets taken. In addition, alprazolam and mirtazapine, which may have been taken in combination, have anticholinergic effects. Anticholinergic effects cause a decrease in peristalsis in the digestive tract, which delays the absorption of the drug. The delayed absorption of lacosamide in this case may have been caused by a combination of these factors. Deslandes et al. reported a case in which Cmax was reached approximately 10 hours after oral administration [20]. Their reported Cmax of lacosamide was 53.9 µg/mL lower than in our case with a half-life of 15-20 hours, which was not significantly different from the reference value. Unlike our case, no concomitant medications inhibiting digestive peristalsis were present. Based on these findings, the primary cause of the delayed increase in lacosamide serum concentration in their case was considered to be delayed absorption due to bezoar formation rather than metabolic saturation. Considering both cases, it is important to recognize that in lacosamide overdose, delayed toxicity may occur due to delayed absorption and metabolism.

To clarify the delay in absorption, it is necessary to confirm the serial changes in serum lacosamide concentration. Similar to our case, Takahashi et al. [11] observed serial changes in serum lacosamide concentration. However, they did not experience a delayed increase in serum lacosamide concentration. The reason for this may be that there was no concomitant use of drugs that caused a decrease in intestinal peristalsis, and gastric lavage and administration of activated charcoal were performed at the time of admission. Gastric lavage may be performed in cases of overdose as a treatment to remove the drug from the body before it is absorbed. In addition, the administration of activated charcoal after gastric lavage has the effect of preventing absorption by adsorbing the drug that could not be removed by gastric lavage in the digestive tract. Therefore, early interventions, such as activated charcoal or gastric lavage, may help mitigate delayed increases in serum levels in suspected overdose cases. In such cases, airway protection such as intubation can prevent aspiration complications, and gastric lavage and activated charcoal administration can be performed safely.

## Conclusions

Severe bradycardia and shock are rare manifestations of lacosamide overdose but may be potentiated by concomitant medications, such as trazodone, that cause QT prolongation and hypotension. Although delayed toxicity due to delayed absorption is a critical consideration, it may be possible to suppress the onset by performing gastric lavage and administering activated charcoal in the initial treatment. In this case, continuous serum concentration monitoring demonstrated a concentration-dependent relationship between lacosamide levels and the onset of severe symptoms. Notably, the risk of severe bradycardia and shock appears to increase when serum lacosamide levels approach 90 µg/mL.
